# Clinical whole Exome Sequencing Reveals Novel Homozygous Missense Variant in the *PMPCA* Gene causing Autosomal Recessive Spinocerebellar Ataxia

**DOI:** 10.12669/pjms.40.10.10474

**Published:** 2024-11

**Authors:** Hala Abubaker Bagabir, Angham Abdulrhman Abdulkareem, Osama Yousef Muthaffar, Bader H. Shirah, Muhammad Imran Naseer

**Affiliations:** 1 Hala Abubaker Bagabir Physiology Department, Faculty of Medicine in Rabigh, King Abdulaziz University, Jeddah, Saudi Arabia; 2 Angham Abdulrhman Abdulkareem Center of Excellence in Genomic Medicine Research, Faculty of Science, Department of Biochemistry, King Abdulaziz University, Jeddah, Saudi Arabia; 3 Osama Yousef Muthaffar Department of Pediatrics, Faculty of Medicine, King Abdulaziz University, Jeddah, Saudi Arabia; 4 Bader H. Shirah Department of Neuroscience, King Faisal Specialist Hospital & Research Centre, Jeddah, Saudi Arabia; 5 Muhammad Imran Naseer Center of Excellence in Genomic Medicine Research, King Abdulaziz University, Jeddah, Saudi Arabia

**Keywords:** Homozygous, *PMPCA*, SCAR2, Developmental Delay, Saudi Arabia

## Abstract

**Background & Objective:**

Autosomal recessive cerebellar ataxias (ARCA) are rare heterogenous neurodegenerative disorders characterized by degeneration of the cerebellum and spinal cord with an early onset before the age of 20 years. PMPCA (MIM: 613036), is a key enzyme in mitochondrial protein processing which is critical for cell survival and growth. Our objective was to investigate Peptidase, Mitochondrial Processing Subunit Alpha (PMPCA) mutations linked with Spinocerebellar ataxia, autosomal recessive 2 (SCAR2).

**Method:**

In the current study, Whole Exome Sequencing (WES) was done followed by Sanger sequencing for the validation of the WES results.

**Results:**

WES results identified a novel homozygous variant, NM_015160.2: c.802C>T p.(Arg268Trp) in *PMPCA* gene. Mutation in this gene leads to progressive cerebellar ataxia with fine motor skills difficulties, intentional tremors, slow slurred speech and learning difficulties in a 12-year-old Saudi patient. WES results were further validated by Sanger sequencing technique.

**Conclusions:**

Identified phenotype in our case was similar as previously described for SCAR2 related conditions. To our knowledge, this is the first reported mutation in *PMPCA* gene leading to SCAR2 in Saudi Arabia. These findings will enrich the scarce literature, further provide a new insight on the role of *PMPCA* gene-related disorders leading to SCAR2 and expand the disease concept. In addition, this will help to establish a database for the disease and its causative factors will further help in controlling diseases resulting from consanguinity in Saudi population.

## INTRODUCTION

Autosomal recessive cerebellar ataxias (ARCA) are rare heterogeneous neurodegenerative disorders involving the central and peripheral nervous systems; it is characterised by degeneration of the cerebellum and spinal cord; although the onset of ARCA can vary widely depending on the specific subtype and is characterised by the early onset before the age of 20 years.^1^,^2^ Clinical presentation, severity and disease progression vary between patients. Clinical phenotype ranging from cerebellar syndromes to sensorimotor neuropathy, ophthalmological disturbances, sensorineural hearing loss, motor developmental delay, involuntary movements, seizures, skeletal anomalies, skin disorders, and cognitive impairment.^1^-^5^ ARCA is challenging during diagnose, however it involves multifaceted approach including a thorough clinical history, neuroimaging, particularly Magnetic resonance imaging (MRI) to detect cerebellar atrophy, and genetic testing.^6^ Currently, there is no cure for ARCA and management primarily focuses on symptomatic relief. However, significant advancements have been made in the molecular and pathological understanding of mechanisms underlying ARCAs. These breakthroughs have unveiled novel diagnostic and therapeutic opportunities, reshaping the landscape of patient care and research in this field.^7^,^8^

Type 2 autosomal recessive spinocerebellar ataxias (SCAR2) (MIM:213200) is type of slow or non-progressive ARCA. In early childhood SCAR2 is categorized by beginning of decreased motor development and ataxic gait.^9^ Autosomal recessive cerebellar ataxias are complex, heterogeneous, disabling inherited neurodegenerative diseases connecting both the peripheral and central nervous systems.^10^ Studies over the past few years have illuminated the genetic underpinnings of SCAR2, linking it to mutations in the *PMPCA* gene.

*PMPCA*, located in 9q34.3 (MIM:613036), is a key enzyme in mitochondrial protein processing which is critical for cell survival. *PMPCA* encodes the alpha subunit of mitochondrial processing peptidase (α-MPP), which cleaves off the N-terminal mitochondrial targeting sequences from precursor proteins upon their import into mitochondria.^11^ The mRNA transcript from PMPCA undergoes translation to produce a protein that forms a heterodimer with the beta subunit encoded by PMPCB.^12^ This heterodimer cleaves targeting sequences from mitochondrial precursor proteins, enabling their proper maturation and function. PMPCA is highly expressed in tissues with significant mitochondrial activity, such as the brain and muscles. Localized in the mitochondrial matrix, it plays a vital role in the mitochondrial protein import pathway, impacting cellular energy metabolism.

Therefore, *PMPCA* mutation causes an abnormal mitochondrial protein processing leading to the accumulation of abnormal nuclear-encoded mitochondrial precursor proteins and ultimately disrupting mitochondrial function. Over the past few years, *PMPCA* mutation was linked with SCAR2. In 2015, Jobling and colleagues were the first to identify variants in *PMPCA* from 17 subjects spanning four different families, all of whom were affected with autosomal recessive non-progressive cerebellar ataxia.^4^

In one of the study French patient has two compound heterozygous mutations (p.Ser96Leu and p.Gly515Arg), while the other 16 Lebanese patients have homozygous missense mutations p.(Ala377Thr).^4^,^13^ These patients had gait ataxia, dysmetria and nystagmus; they presented with hypotonia and delayed gross motor, which gradually improved. Similarly, two French Canadian brothers with SCAR2 caused by a homozygous mutation in *PMPCA* p.(Val256Met), presented with different severity of gait impairment, dysarthria, dysmetria and distal atrophy without intellectual deficiency.^3^ On the other hand, one reported extremely severe clinical features with diffuse parenchymal volume loss as viewed on brain magnetic resonance imaging (MRI) with PMPCA variants. More recently, Takahashi et al reported a novel compound heterozygous PMPCA variants, c.667C > T p. (Arg223Cys) and c.853del p. (Asp285llefs*16), in a Japanese girl of 15 years old, who presented with a severe phenotype of a progressive developmental delay, cerebellar ataxia, and extrapyramidal symptoms.^14^

Recently we have reported novel variant in *CWF19L1* gene in a family with late-onset autosomal recessive cerebellar ataxia 17 and a novel mutation in *ATM* gene in a female with ataxia telangiectasia.^15^,^16^ In another study, we reported autosomal recessive cerebellar ataxia with spasticity due to a rare mutation in *GBA2* gene.^17^ Further we also reported novel mutation in *CACNA1A* gene with episodic ataxia type 2 and rare mutation in the *SETX* gene in a Saudi patient.^18^,^19^

SCAR2 is a rare neurodegenerative disorder, and is thus for not reported in Saudi Arabia. In the current study, we report a novel homozygous variant in *PMPCA* gene in a 12-year-old Saudi boy with progressive cerebellar ataxia with frequent falls, slow slurred speech, learning difficulty and fine motor skills difficulties started during his childhood. The identified mutation will add value to the literature of the related disease, provide a new insight into PMPCA gene-related disorders and expand the disease concept that will further help to tackle the causative factors leading to SCAR2.

## METHODS

### Clinical report of the patient:

The current study reported a 12-year-old male IV-1 proband with SCRA2 who presented since early childhood with progressive cerebellar ataxia, fine motor skills difficulties, slow slurred speech and learning difficulty without intellectual deficits. On examination, the patient was conscious and oriented with no dysmorphic features. Ophthalmic examination revealed normal ocular movement no squint or nystagmus, and normal pupillary reflex in addition to the normal cranial nerves function. Sensory examination was normal. Motor examination showed good power, dystonia, +3 deep tendon reflex, positive dysdydokinesia and intentional tremors with bilateral dysmetria. MRI examination of the patient showed abnormal intensity of the posterior limb of bilateral internal capsule, crus cerebri, pons and medulla with normal cerebellum. The patient is a product of three generations of consanguine marriages. The grandparents and the parents were first-degree cousins with three children. After the clinical details and phenotypical analysis WES was recommended for the affected proband to understand the disease genetics.

### Ethical approval and Sample collections:

The study received approval from the local ethical committee at the Center of Excellence in Genomic Medicine Research, King AbdulAziz University in Jeddah (Ref# 013-CEGMR-02-ETH). Written informed consent for laboratory and genetic tests was obtained from the patient’s legal guardians. This research work done in CEGMR, King AbdulAziz University Hospital in 2022 and 2023. Blood samples from the patient and their family were taken post-DNA extraction. The experiments adhered to international guidelines following the Declaration of Helsinki 2013. DNA was extracted from the patient’s blood, and preserved in EDTA tubes from Roche Life Science, following previously established methods. The DNA concentration and quality were assessed using NanodropTM 2000/2000c spectrophotometers.

### Whole exome sequencing (WES):

After the DNA from the patient WES was done for the coding region and splice site junctions of the diseased genes. Illumine NextSeq state of the art instrument with 2x76 paired end reads was used. Reference sequences from hg19, GRCH37/UCSC was used. All sequence alterations are defined under the guidelines of Human Genome Variation society nomenclature rules. Various bioinformatics tools were used for variant calling. The products were sequenced on an Illumina NextSeq instrument with 2 × 76 paired-end reads as previously described.^20^,^21^

After WES, (FASTQ) files were generated and then these files were further converted to BAM than to variant call format (vcf) files having all identified variants. Identification of variants those leading to the disease phenotype was established through rare, ulta rare, novel (MAF+0.01%) frequency, homozygosity or heterozygosity settings, structure changes and functional (predicted damage by Polyphen/SIFT), genomic position, pathogenicity, protein damaging effects etc. Moreover, online available tools and filters were applied using different Bioinformatics application, reference sequence from GRCh37 database were used.

The list of obtained variants were filtered to find out the disease linked with the identified variants in public databases, such as Genome Aggregation Database (gnomAD), for allele frequencies <5.0% in the http://gnomad.broadinstitute.org/), and nonsense, frameshift, and splice-site variants in disease-associated genes with a minor allele frequency ≤1.0% were observed in gnomAD in dbSNP (https://www.ncbi.nlm.nih.gov/SNP), HapMap (https://www.genome.gov/international-hapmap-project), 1000 Genomes Project (http://www.internationalgenome.org).^22^-^24^ We also filter candidate SNPs with the following criterion: snp quality ≧20, sequencing depth ≧4-fold, estimated copy number ≦2 and the distance between two SNPs is larger than five insertions and deletions (indels) in the exome regions were identified through the sequencing reads as previously done.^25^

Various lines of computational evidence support a deleterious effect of the gene and its product such as (evolutionary, splicing impact, conservation) etc. Classification of identified variants were done by using the American College of Pathologists (ACMG/AMP) criteria.^26^ HGVS nomenclature (https://varnomen.hgvs.org/) was used to report the variants. Deleterious effects and abnormalities were also identified using *in silico* analysis for structure and function of the identified variant leading to the disease. In silico studies were also done for missense variants to predict the effect of amino acid substitutions on protein structure. Mutation Tester (http://www.mutationtaster.org/) also predicted the variant as disease causing variant. Implementation of WES technique improves the screening and identification of novel and causative genetic variants, which aid in the diagnosis of genetic disorders.

### Sanger sequencing:

After the WES the identified variant, it was further validated by using the gold standard Sanger sequencing technique to verify a novel homozygous missense variant in *PMPCA* gene. We designed the targeted primers using online free software primer 3 for PCR and for Sanger sequencing technique for all affected and normal members of the family. For PCR and sequencing, the sets of the targeted primers were designed by using the online primer 3 program. Forward primer sequence was PMPCAF:5’-ACCTGTGTCTGTGGCTCTTC-3’ and the reverse primer sequence was PMPCAR:5’-GCAGTCAACACCTATCACCC-3’. After sequencing the files were obtained from the AB1 sequencing unit. The obtained files were aligned with the reference sequence using the BioEdit software.

### Computational structural analysis of mutants:

The *PMPCA* sequence retrieved from the Uniprot database. SWISS-MODEL2 was used to produce the homology model. Models were manually inspected, and mutations evaluated, using the Pymol program (pymol.org).

## RESULTS

**Fig.1:**
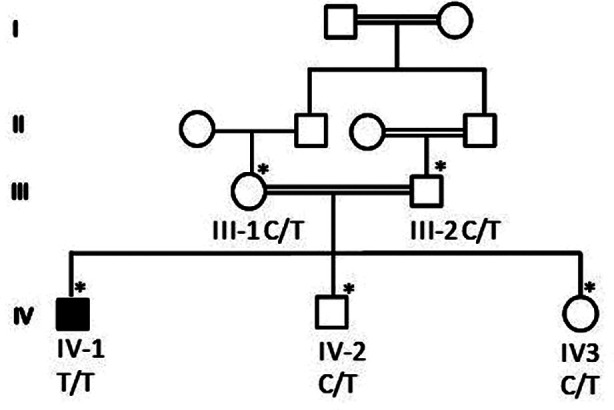
Shows the pedigree of the family. IV-1 is the affected boy. *mean the available samples for the study.

**Fig.2:**
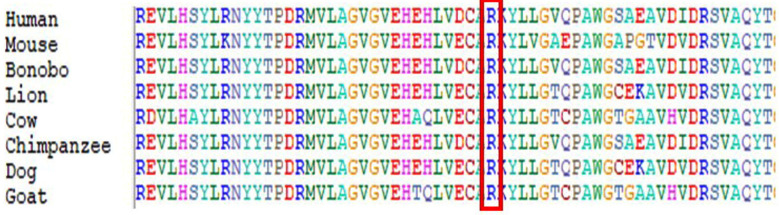
Representative electropherogram of PMPCA gene. Sanger sequencing results show that the III-1 and III-2 parents are the heterozygous carries having C/T on both alleles while the probandIV-1 showing a novel homozygous variant c.802C>T (p.Arg268Trp) in identified PMPCA gene, wherase IV-2 and IV-3 were normal wild type heterozygous C/T.

Whole Exome Sequencing (WES) results identified a novel homozygous variant, NM_015160.2: c.802C>T p. (Arg268Trp) in *PMPCA* (MIM:613036) gene. The patient was homozygous for the *PMPCA* variant c.802C>T where p.(Arg268Trp) amino acid was changed in the protein. The parents were heterozygous carriers who were first-degree cousins, as depicted in the pedigree Fig.1. Sanger sequencing chromatogram in Fig.2 showed that both the parents III-1 and III-2 are heterozygous carriers having C/T on both alleles. The affected child is a 12 years old boy IV-1 was homozygous having T/T and two of his siblings IV-2 (a boy) and IV-3 (a girl) were heterozygous carrier both having C/T alleles while the wild type control has C/C base pair on both allele. Further validation was performed by using 100 normal control samples from Saudi Arabia to confirm that the identified variant is not present in the normal population. To our knowledge, this variant has not been previously reported in the literature, and this is the first reported SCAR2 case in Saudi Arabia.

Moreover, the variant is not reported in gnomAD exomes and 1000 genomes. There is a physiochemical difference between arginine and tryptophan. The clinical and molecular assessments consistently diagnosed the *PMPCA* (MIM:613036). PP1 co-segregation with disease in multiple affected family members in a gene definitively known to cause the disease. Multiple lines of computational evidence support a deleterious effect on the gene or gene product (conservation, evolutionary, splicing impact) etc.

**Fig.3:**
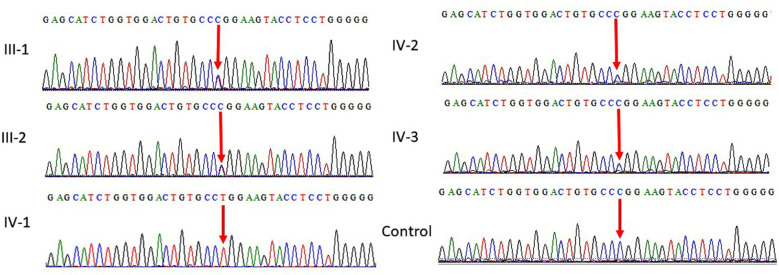
The amino acid arginine at residue 268 in human PMPCA is highly conserved across many species, including mouse, Bonobo, Lion, Cow, Chimpanzee, Dog and Goat.

**Fig.4:**
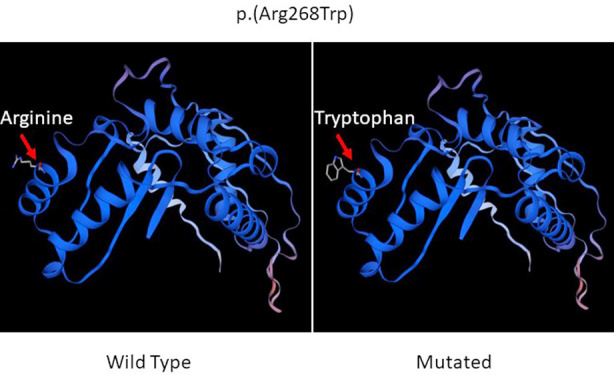
Structural analysis of the Arg268Trp variant in PMPCA. Mitochondrial-processing peptidase subunit alpha AlphaFold DB model of MPPA_PONAB (gene: PMPCA, organism: Pongo abelii (Sumatran orangutan) (Pongo pygmaeus abelii)) using Swiss model. A, showing the wild type normal while B, showing the mutated protein.

Furthermore, the results of in-silico tools, commercially and publicly available, are used to aid in interpreting sequence variants identified the variant as disease-causing. Moreover, protein alignment of the identified variants leading to the protein change was studied. It showed that sequence of Arginine is absolutely conserved in the region between different species throughout evolution including mammals, reptiles and nematode as shown in (Fig.3). *PMPCA* gene encode substrate recognition and binding subunit sites of the essential mitochondrial processing protease (MPP), which is required for maturation of the majority of mitochondrial precursor proteins. This substitution of arginine to tryptophan would result in disruption of enzyme activity and further this substitution may increase in net negative charge of the protein through the inability of the uncharged tryptophan side-chain to polarize the substrate carbonyl bond. Structure of wild type and mutated proteins are shown in Fig.4.

## DISCUSSION

This study reported the first case of SCAR2 from a Saudi family where a novel homozygous variant, c.802C>T p.(Arg268Trp) in PMPCA was identified. Our case presented the typical SCAR2 phenotype with additional motor skill issues, uncommon in SCAR2*. PMPCA* encodes for α-MPP, which is essential for precursor cleavage from mitochondrial proteins to attain their function.^11^ Disruption of α-MPP is attributable to the accumulation of abnormal mitochondrial precursor proteins, which disturb mitochondrial function and stop cell viability. Any mutation in *PMPCA* variants changeMMP expression and decrease frataxin (a nuclear-encoded mitochondrial protein) which play an important role in MMP cleavage pathways, for the biosynthesis of iron-sulfur cluster in cells.^27^ Friedreich ataxia is caused by GAA triplet repeat expansions in the first intron of the frataxin gene.^28^ Iron accumulation in cerebellum and basal ganglia was noted clinically in Friedreich ataxia patients.^29^ This neurogenetic pathophysiology may explain why PMPCA gene variants are important to cause the phenotype of extrapyramidal symptoms as well as cerebellar ataxia and suspected iron accumulation in brain imaging.

In recent years, *PMPCA* has been found to cause SCAR2, with typical phenotype of gait ataxia, dysmetria, dysarthria, nystagmus and intellectual disability in most cases.^4^,^14^ Joshi et al, reported a large family with two members presenting with *PMPCA* mutation; both patients presented at the age of six months with severe developmental delay and failure to thrive with ocular manifestations including bilateral ptosis, and ophthalmoplegia; one died due to severe hypertrophic left ventricular cardiomyopathy and liver failure.^30^

Our case presented with the typical phenotype in addition to fine motor skills difficulties, intentional tremors, learning difficulty and slurred speech; however, the patient had neither intellectual disability nor nystagmus. This might be justified by the normal cerebellum examined by MRI in this current study, whereas other studies have shown mainly atrophied cerebellum.^3^,^4^,^14^,^30^ Furthermore, Takahashi et al, also reported the involvement of bilateral globus pallidum and substantia nigra along with cerebellar cortex and vermis in which the patient presented with cerebellar symptoms in addition to extrapyramidal symptoms with cogwheel rigidity and dystonic features.^14^ Full characterisation of the SCAR2 due to *PMPCA* mutation based on the literature are summaries by Takahashi et al.^14^

*PMPCA* variants have been shown to follow a mild and non-progressive course of cerebellar ataxia and developmental delay.^4^ However, Choquet et al. presented a case of two brothers with considerably different severity; one showed a slowly progressive spinocerebellar with learning difficulties during adolescence, while his sibling had a severe form of the disease with hemidystonia and neurosensorial hearing loss at the age of five years.^3^ Severe and progressive presentation of the disease was also reported from a Japanese 15-year-old girl during infancy.^14^ Brain MRI reported extremely severe clinical features with diffuse parenchymal volume loss in the patients having *PMPCA* variants.^30^

In another study the patient also exhibited poor body movements during sleep, characteristic findings seen in Segawa disease, which suggests low dopaminergic activity in the nigrostriatal pathway.^31^ Similarly, the patient from the current study presented at the age of 12 with regression of health started in early childhood with tremors, progressive gait abnormality and fine motor skills difficulties with preserved intellectual function.

In the current study, validated WES results by Sanger sequencing identified a homozygous variant, NM_015160.2: c.802C>T p.(Arg268Trp) in *PMPCA* gene in a 12 year-old-male patient, proband IV-1. The parents, who were first-degree cousins, were heterozygous carriers and his brother IV-2 and sister IV-3 are heterozygous carrier for the disease. *PMPCA* gene variants from previous studies are summarised in Table-I.

**Table-I:** Characteristics and genetic studies in our patient reported with PMPCA gene variants known so far in the literature.

Serial no.	Mutation	Protein	Progressive	Regression	Walk	Language acquisition	Intellectual disability	Dysarthria	Ataxic gait	Nystagmus	Rigidity/ tonus	Dystonic feature	Brain MRI	Reference
1	c.667C > T / c.853del	p.Arg223Cys / p.Asp285llefs*16)	+	+	+	+	+	+	+	+	Cogwheel ridigity / hypertonic	+	Atrophy of cerebellar vermis and hemispheres with T2 hyperintense signal at cerebellar cortex. Low intense areas at bilateral globus pallidus and substantia nigra on SWI	[14]
2	c.1129G >A	p.Ala377Thr	+	+	+	+	+	+	+	+	Hypotonic	N	Cerebellar vermis and bilateral hemisphere hypoplasia, dilated 4th ventricle, and large cisterna magna	[4]
3	c.1129G >A c.287C > T/	p.Ala377Thr/ p.Ser96Leu /	+	+	+	+	+	+	+	+	Hypotonic	N	Cerebellar atrophy with vermian predominance	[13]
4	c.1543G > A/ c.766G > A	p.Gly515Arg /p.Val256Met	Slowly	-	+	+	-	+	+	+	-	Hemidystonia	Atrophy of cerebellar vermis and hemispheres	[4]
5	c.553C > T	p.Arg185Trp	+	+	+	-	+	NA	+	N	Spastic	+	Cerebellar atrophy and bilateral symmetrical hyperintensity in the striatum	[36]
6	c.1066G>A/ c.1129G > A	p.Gly356Ser) / p.Ala377Thr)	+	+	-	-	+	NA	N	+	Hypotonic	N	Progressive generalized brain atrophy, with diffuse parenchymal volume loss and areas of gliosis and severe ventriculomegaly involving both of the lateral ventricles as well as the third and fourth ventricles Marked cerebellar and mild cerebral atrophy	[30]
7	c.633+1G>A	-	+	-	+	-	+	NA	+	+	-	N	CA Cerebellar cortical hyperintensity	[35]
8	c.802C>T	p.Arg268Trp	+	+	+	+	+	NA	+	+	Hypotonic	N	CA Cerebellar cortical FLAIR hyperintensity	Present Study

N, not mentioned; NA, not available due to its severity.

Furthermore, SCAR2, due to *PMPCA* mutations, is an autosomal recessive genetic disease. Therefore, consanguinity is a major player in the disease inheritance. Having said that, the reported cases were both nonconsanguineous and consanguineous parents,^3^,^4^,^14^,^30^, the patient from the current study, however, was a product of three generations of consanguine marriages. Consanguinity is an inherited practice in Saudi Arabia which increases the prevalence of genetic diseases. WES technique is dedicated to identifying genetic defects in patients with suspected genetic disorders. The diagnostic capability of WES ranges between 25 to 35%, with trio analysis yielding a maximum of 40%.^32^-^36^ Identifying pathological genetic variants will help in genetic counselling and aid in developing therapeutic strategies against the disease through prenatal testing.

### Limitations:

Further functional studies and collection of the patients with *PMPCA* gene variants would be important steps to understand the pathophysiology of the disease.

## CONCLUSION

This study reported the first case of SCAR2 from a Saudi family where a novel homozygous variant, c.802C>T p.(Arg268Trp) in *PMPCA* was identified. This case is reporting a rare disease and a rare genetic variant related to the *PMPCA* gene in Saudi Arabia will further expanding the knowledge about the mutation spectrum of *PMPCA*, helping to address SCAR2 in the future. The use of WES testing from patients, especially products of consanguineous marriages, along with a strong family history, are highly advisable.

### Authors’ Contribution:

**MIN, HAB and OYM:** Idea, methodology and Review.

**AAA, MIN:** Experiment and Data analysis, Critical Review.

**HAB, BHS:** Preparation of original draft, coresponsibility and integrity of the study.HAB, BHS and MIN: Manuscript correction and editing.

**MIN:** Manuscript correction and final editing for submission.

All authors have read the final manuscript and are responsible for the integrity of the study.
